# A Case Report of Neuro-Behçet Syndrome: Frequent Neurological Manifestations Concurrent With Life-Threatening Illnesses

**DOI:** 10.7759/cureus.54664

**Published:** 2024-02-21

**Authors:** Omar Ballut, Mayas M Almahi, Banan S Alghamdi, Najla K Alzahrani, Maali A Alghamdi

**Affiliations:** 1 Internal Medicine, King Fahad Hospital, Al-Baha, SAU; 2 Internal Medicine, Royal College of Physicians of Edinburgh, Edinburgh, GBR; 3 Medical School, Al-Baha University, Al-Baha, SAU

**Keywords:** septic shock, infective endocarditis, neuro-behçet syndrome, neurological involvement in behçet disease, behçet disease

## Abstract

​​​​Behçet disease (BD) is a recurrent, multisystemic autoimmune vasculitis that affects both small and large vessels. A combination of neurological signs and symptoms in BD is called neuro-Behçet syndrome (NBS). We present the case of a 31-year-old male diagnosed with chronic progressive NBS who presented with multiple relapsing episodes concurrent with infective endocarditis due to intravenous drug abuse, drug-induced hepatitis, acute kidney injury, and septic shock that is not related to BD. Neurological relapsing episodes were treated with steroids azathioprine and colchicine. At the same time, concurrent illnesses were managed appropriately. Infective endocarditis needed valve replacement surgery, and sepsis was treated with selected antibiotics. Fortunately, the patient's brain images and laboratory investigation improved accordingly. On average, patients with parenchymal neuro-Behçet syndrome (P-NBS) have a poor prognosis; within 10 years of diagnosis, 50% of those patients are severely disabled as our patient who became aphasic and quadriplegic.

## Introduction

Behçet disease (BD) is a rare, autoimmune, recurrent, inflammatory, multisystemic disorder of unknown etiology [1]. Initially, Hulusi Behçet, a Turkish dermatologist, defined BD as recurrent oral and genital ulcerations and ocular inflammation in 1937 [2].

Neurological involvement in BD, called neuro-Behçet disease (NBD), has to be differentiated from multiple sclerosis (MS), neuromyelitis optica spectrum disorders (NMOSD), stroke, cerebral venous sinus thrombosis (CVST), spinal cord disorders, presenile dementia, aseptic meningitis, and other common neurological diseases. So, diagnosis and management of the disease could be challenging [3].

As there is limited data in the literature, we present a unique case of a patient with NBD who presented with recurrent episodes of central nervous system (CNS) involvement concurrent with non-neurological BD in the presence of BD-unrelated signs and symptoms of concurrent diseases which are difficult to differentiate from BD systemic involvement.

## Case presentation

**Figure 1:**
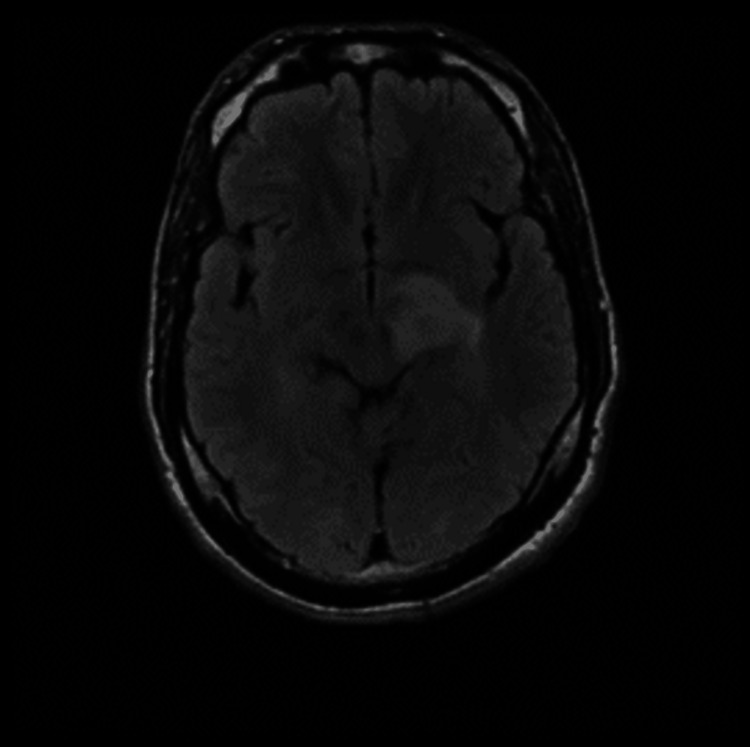
Brain MRI revealed abnormal signal intensity lesion seen at the left thalamus, posterior limb of the internal capsule, and left superior cerebellar peduncle reflecting hyperintense signals at FLAIR WI. FLAIR WI: fluid-attenuated inversion recovery-weighted image

A 31-year-old male presented to the emergency room (ER) with progressive weakness of the right upper and lower limbs associated with slurred speech and severe generalized headache. He had a history of severe headache episodes with blurred vision four months ago, which resolved with analgesics, and a long history of both oral and genital ulcers. On examination, he was conscious, alert, and oriented. His Glasgow Coma Scale (GCS) was 15/15. He was vitally stable, with a clear chest and a soft and lax abdomen. Oral and scrotal ulcers were found. Strength of the right upper and lower limbs was estimated as grade 4, while grade 5 was estimated for both left upper and lower limbs. Cranial nerves were intact, sensations were all intact bilaterally, and there was an abnormal left plantar reflex. Laboratory investigations were all within normal limits as shown in Table 1. Furthermore, the pathergy test was positive, along with positive results for both HLA-B50 and HLA-B51 lab tests. A brain MRI was performed and showed left hyperintense lesions (Figure 1).

**Table 1:** Laboratory investigations were normal. WBCs: white blood cells; CK: creatine kinase; CK-MB: creatine kinase MB; INR: international normalized ratio

Laboratory investigation	
WBCs	5.78x10^9^/L
Hemoglobin	13.90 g/dL
Platelet count	228x10^3^/uL
Serum albumin	39 g/L
Serum sodium	138 mmol/L
Serum potassium	3.60 mEq/L
CK	1256.50 IU/L
CK-MB	25.40 IU/L
INR	1

**Figure 2:**
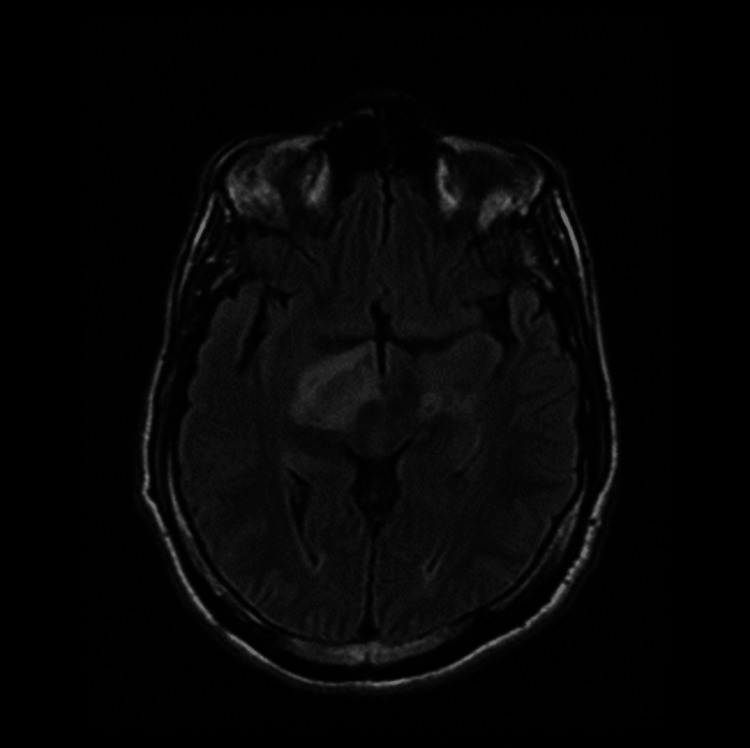
Brain MRI revealed abnormal signal intensity lesion seen at the right thalamus and right superior cerebellar peduncle reflecting hyperintense signals at FLAIR WI. FLAIR WI: fluid-attenuated inversion recovery-weighted image

Therefore, this patient met the International Study Group (ISG) criteria, and he scored 6 regarding the International Criteria for Behçet Disease [4]. The diagnosis of BD with neurological involvement was confirmed based on the patient's presentation of neurological signs and symptoms, supported by positive findings on brain MRI. Furthermore, thorough evaluation ruled out other potential causes such as structural abnormalities and infectious conditions, reinforcing the likelihood of BD as the underlying etiology. The patient was started five days of therapy on 1 g IV methylprednisolone once daily (OD) and discharged on 60 mg prednisolone OD with slow tapering, 50 mg azathioprine twice daily (BID), and 0.5 mg colchicine BID. Two months later, he presented to the ER complaining of left upper and lower limb weakness, dysarthria, and blurred vision that started a day ago with generalized fatigue. The physical examination revealed a decrease in the strength of the left upper and lower limbs of grade 3 and dysarthria, otherwise unremarkable. Laboratory investigations showed no abnormalities as displayed in Table 2. A brain MRI was done and showed right hyperintense lesions (Figure 2).

**Table 2:** Normal laboratory investigation WBCs: white blood cells; ESR: erythrocyte sedimentation rate; BUN: blood urea nitrogen

Laboratory investigation	
WBCs	11x10^9^/L
Hemoglobin	14 g/dL
Platelet count	245x10^3^/uL
ESR	2 mm/hr
Creatinine	68 micromoles/L
BUN	4 mmol/L

He was on pulse 1 g IV methylprednisolone OD for five days. He shifted to a high dose of prednisolone 60 mg per os (PO) OD for six months. He continued on azathioprine as the best disease-modifying drug, together with 2-3 mg/kg of folic acid to avoid anemia. 

After two months, he was admitted to the hospital due to left-side weakness and a history of IV amphetamine abuse. The patient was confused with a GCS of 11/15, tremulous, overactive, aggressive, feverish, and tachycardic, but otherwise vitally stable. On auscultation, there was a systolic murmur at the left lower sternal edge grade 3/6. Laboratory investigations showed a slightly increased white blood cell count of 11.45x106 with neutrophilia of 77% and an elevated creatine kinase (CK) of 1183 U/L. Liver function tests showed a normal albumin level of 30.2 g/L, an elevated alanine aminotransferase (ALT) and aspartate aminotransferase (AST) of 337 U/L and 1119.6 U/L, respectively, as well as a creatinine of 154 µmol/L. Electrolytes were normal except for sodium which was 131 mEq/L, urine analysis showed gross hematuria, +3 albumin, +3 glucose, and 15-20 pus cells, and the cardiac profile showed a lactate dehydrogenase (LDH) of 4911 IU/L and CK-MB of 823 IU/L. Urine and blood cultures were positive for methicillin-resistant *Staphylococcus aureus*. An electrocardiogram (ECG) showed a right-axis deviation and Q wave on leads V4-V6 with pause pulse. An echocardiogram revealed left ventricle dysfunction with a normal ejection fraction of 60% and no regional wall motion abnormality and grossly dilated right ventricle and atrium with giant mobile vegetation on both leaflets of tricuspid valve larger on the lateral wall leaflet with deterioration of valve structure suggesting a severe tricuspid regurgitation and pulmonary arterial systolic pressure of 100 mmHg. Therefore, infective endocarditis (IE) was diagnosed according to the Duke criteria, and by two major criteria, there were two positive blood cultures for methicillin-resistant *Staphylococcus aureus* and echocardiogram findings.

Abdominal ultrasound showed a mildly enlarged liver with diffused bright echotexture changes suggesting a fatty liver, and an isoechoic lesion was noted at the sub-scapular area of the left kidney. He has a long history of IV and oral amphetamine abuse which reaches four pills per day done multiple times, and he was already diagnosed with substance-induced psychosis by a psychiatrist; therefore, by the exclusion of autoimmune hepatitis and viral hepatitis after negative results for hepatitis B surface antigen and HIV AG/AB combination, drug-induced hepatitis caused by IV amphetamine abuse was diagnosed. Moreover, sepsis and amphetamine-induced acute kidney injury were diagnosed. He was started on methylprednisolone 40 mg IV and antibiotics. The clinical symptoms improved gradually with regression of neurological and cardiac manifestations, and he was transferred to another hospital where an open heart surgery for tricuspid valve replacement was successfully performed.

**Figure 3:**
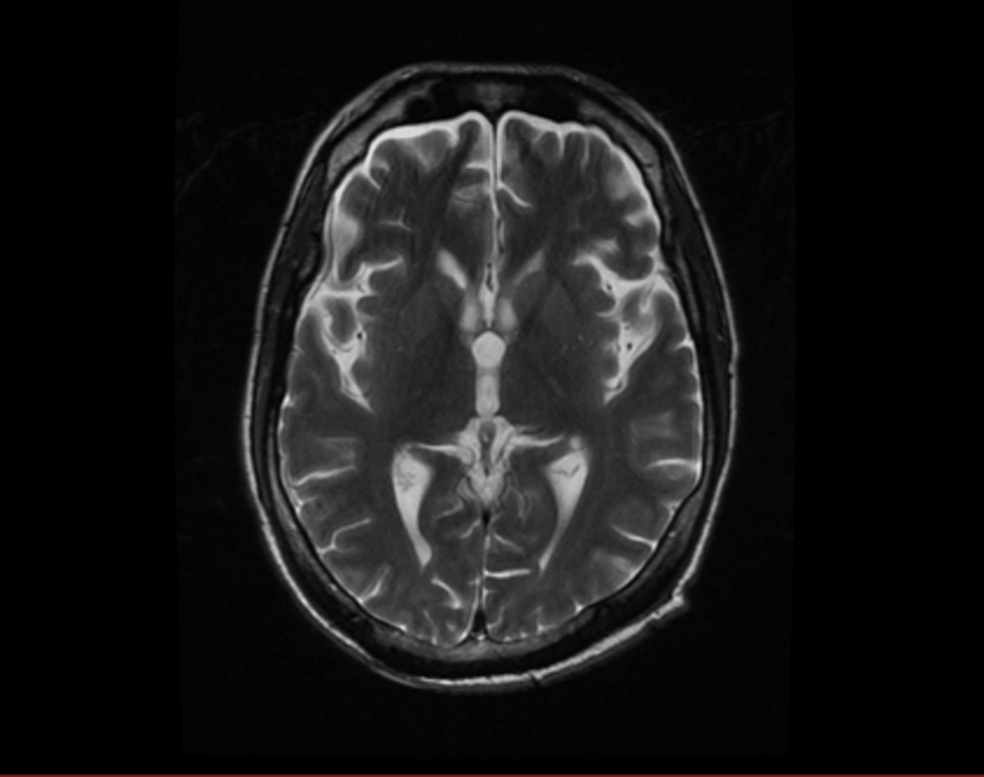
Brain MRI T2 showing significant resolution of the hyperintense lesions in the left internal capsule, both thalami and brainstem.

Four months later, he was referred from outside as a case of septic shock; he was complaining of refractory fever for a week with recurrent choking during feeding, cough, vomiting, and diarrhea. On examination, he had been bedridden since the valve replacement operation and looked ill, wasted, and conscious but aphasic with excessive yawning; his blood pressure was 80/50 mmHg with no vasopressor used; he was tachycardic with 102 bpm, tachypneic with 29 breaths per minute, and afebrile; and his SPO2 was low: 82% on room air. On auscultation, coarse crepitations were heard all over his chest with transmitted sounds. Neurological examination was significant of quadriplegia with stiffness and hyperreflexia with the strength of no more than grade 1 in the lower limbs; he had decubitus ulcer of grade 2. Laboratory showed leukocytosis of 24x109/L and hypokalemia of 2.89 mmol/L, sputum culture was positive for multi-drug-resistant *Klebsiella pneumoniae*, and there was mixed growth of contaminants in blood culture. Venous blood gas (VBG) showed compensated metabolic acidosis: pH of 7.37, PCO2 of 24 mmHg, HCO3 of 17 mEq/L, and lactic acid of 2.5 mmol/L. Sinus tachycardia on ECG and chest X-ray showed bilateral peripheral infiltration, especially in the left lower zone. The echocardiogram showed a healthy tricuspid tissue valve with no vegetation and an ejection fraction of 60%. Fluid intake and output were in positive balance. A brain MRI was performed and revealed significant improvement (Figure 3).

However, he was diagnosed as a case of sepsis due to a urinary tract infection (UTI) and multi-drug-resistant *Klebsiella pneumoniae*. He was transferred to the ICU as GCS dropped to 9-10/15, along with a decrease in oxygen saturation. So, he needed to be kept on higher oxygen flow pressure (on FM 5L); his general condition was improved as injection of colistin was started as a 9 million loading dose and then 4.5 million units IV BID for five days with daily renal function test and daily renal dose adjustment and dexamethasone 6 mg IV OD 5/7. After he was hemodynamically stable and could maintain a proper oxygen saturation on room air with no signs of infection and a GCS of 15/15, he was transferred back to a regular ward. He was discharged on colchicine 0.5 mg BID PO 7/7, azathioprine 50 mg BID PO 7/7, and prednisolone 35 mg OD PO 2/7 and then 30 mg for 7/7 which was reduced by 5 mg every week. 

## Discussion

This case report highlights the intricate management of a 31-year-old male presenting with progressive weakness, slurred speech, severe headache, and a history of oral and genital ulcers, ultimately diagnosed with BD with neurological involvement. The patient's clinical course was complicated by recurrent neurological manifestations, IE, drug-induced hepatitis, acute kidney injury, sepsis, and multi-drug-resistant infections.

Interestingly, the resolution of neurological lesions observed in imaging studies following treatment with IV methylprednisolone followed by oral prednisolone, azathioprine, and colchicine highlights the potential efficacy of aggressive immunosuppressive therapy in managing neurological manifestations of BD. In the medical literature, there are several reported cases of BD with neurological involvement and associated complications, highlighting the complexity and variability of this condition. These cases often emphasize the challenges in diagnosis and management and the potential for various systemic manifestations.

Drawing parallels to a case reported in the literature, a 20-year-old male presented with a spectrum of neurological symptoms, including headache, vision loss, weakness, and aphasia, culminating in a diagnosis of neuro-Behçet syndrome (NBS). Treatment involving steroids yielded significant improvement. While both cases share commonalities in neurological manifestations and a history of oral and genital ulcers, the diverse outcomes and complications underscore the intricate clinical trajectory and management complexities associated with autoimmune disorders like BD [5].

Another case of a 29-year-old female with BD presented with progressive weakness, sensory deficits, and urinary incontinence attributed to spinal cord involvement. MRI confirmed spinal cord lesions consistent with NBD. Prompt initiation of corticosteroids, azathioprine, and intravenous immunoglobulin (IVIG) resulted in notable improvement in neurological symptoms and radiological findings, underscoring the significance of early recognition and aggressive treatment in preventing irreversible neurological damage [6].

Additionally, there are cases reporting cardiovascular complications in BD, including arterial and venous thrombosis, aneurysms, and endocarditis. These complications often necessitate specialized management strategies, including anticoagulation therapy, surgical interventions, and close monitoring for infectious complications [7].

Distinct studies highlight cases where NBD and BD are exacerbated due to an active coronavirus disease 2019 (COVID-19) infection, suggesting a potential link between infections and NBD exacerbations. All these cases emphasize the challenge of effectively managing the immune response. The use of aggressive immunosuppressant therapy in these instances, resulting in improvement, underscores the crucial need for a nuanced understanding of immune dynamics in NBD exacerbations influenced by various factors, including infections. This awareness lays the foundation for developing targeted therapeutic approaches that acknowledge the multifaceted nature of these immune-mediated conditions [8,9].

As our patient struggled with addiction, after the literature review, few case reports have connected NBS and psychiatric disorders. Depression and anxiety are the most consistently documented psychological conditions in BD and are found at relatively similar levels in both BD and NBD patients. However, there is not enough data to support that BD will increase the addiction rate [10].

This case underscores the importance of a multidisciplinary approach involving rheumatology, neurology, cardiology, infectious diseases, and critical care specialties in managing complex cases of BD with neurological involvement and associated complications. Long-term management strategies focusing on immunosuppression, infection control, and addressing underlying risk factors are essential in optimizing patient outcomes and preventing disease relapse. Close monitoring for disease activity, treatment response, and complications is paramount in guiding treatment decisions and improving overall prognosis.

The prognosis in NBD varies widely depending on several factors, including the type and severity of neurological involvement, frequency of relapses, and effectiveness of treatment [11]. Isolated intracranial hypertension and dural venous sinus thrombosis often respond well to therapy, leading to a favorable prognosis with minimal long-term complications [12]. However, patients similar to our case with parenchymal neuro-Behçet syndrome (p-NBS) typically experience a more progressive and debilitating course, often resulting in severe neurological deficits and disability [13]. The presence of systemic symptoms, frequent relapses, and concurrent life-threatening diseases further contribute to poor outcomes and increased mortality risk [14].

## Conclusions

This case report underscores the intricate management challenges involved in treating BD with neurological involvement and its associated complications. The patient's journey highlights the recurrent nature of the disease, the potential efficacy of aggressive immunosuppressive therapy in managing neurological manifestations, and the importance of a multidisciplinary approach in optimizing patient care. Despite the complexities and variability of NBD, ongoing research and clinical collaboration offer hope for enhanced therapeutic approaches and improved patient prognosis in the future.
